# Airborne Suspended Particulate Matter and the Prevalence of Allergic Conjunctivitis in Japan

**DOI:** 10.7759/cureus.53292

**Published:** 2024-01-31

**Authors:** Tatsuya Mimura, Takamichi Ichinose, Ken-ichiro Inoue, Yasuhiro Yoshida, Hiroshi Fujishima

**Affiliations:** 1 Ophthalmology, Teikyo University School of Medicine, Tokyo, JPN; 2 Department of Health Science, Oita University of Nursing and Health Sciences, Oita, JPN; 3 Graduate School of Nursing, University of Shizuoka, Shizuoka, JPN; 4 Department of Immunology and Parasitology, School of Medicine, University of Occupational and Environmental Health, Fukuoka, JPN; 5 Ophthalmology, Tsurumi University, Yokohama, JPN

**Keywords:** environment, suspended particulate matter, outpatient attendance, allergic conjunctivitis, air pollution

## Abstract

Background

This study aimed to examine the association of suspended particulate matter (SPM) with outpatient attendance for allergic conjunctivitis.

Methodology

The information on air pollution, encompassing total hydrocarbons, non-methane hydrocarbons, methane, carbon monoxide, nitrogen oxide, nitric oxide, oxidants, and SPM alongside data concerning daily weather conditions such as temperature, wind speed, and humidity, was gathered. Subsequently, the weekly mean values for outpatient visits, air pollution, and weather parameters were computed.

Results

The number of outpatient visits for allergic conjunctivitis was significantly associated with SPM levels (r = 0.70, p = 0.0037), oxidant levels (r = 0.70, p = 0.0038), wind speed (r = 0.48, p = 0.0472), and humidity (r = 0.77, p = 0.0009) from January to March, as well as SPM levels (r = 0.53, p = 0.0309) and carbon monoxide (r = 0.56, p = 0.0230) from April to June. Multivariate analysis showed that SPM (odds ratio = 1.37, p = 0.0161) and wind velocity (odds ratio = 1.52, p = 0.0038) were significant predictors of the number of outpatient visits from January to December.

Conclusions

SPM levels were the only independent predictor of outpatient visits for allergic conjunctivitis, suggesting that SPM contributes to the pathophysiology of this condition.

## Introduction

Tokyo is a typical example of a megacity. Megacities have a global impact on air quality because their automobiles, trucks, buses, and motorcycles are commonly responsible for a considerable amount of air pollution. In December 2000, the Tokyo Metropolitan Assembly passed an ordinance regulating diesel-powered vehicles [1]. In October 2003, the Tokyo Metropolitan Government began to regulate emissions from diesel-powered trucks and buses in cooperation with eight local governments of the Kanto district near Tokyo. After these legal restrictions on the use of diesel vehicles came into force, air pollution gradually improved in Tokyo [2,3].

However, particulate air pollution has increased over the past few decades in many cities around the world due to more motor vehicle traffic and industrial emissions. In Japan, the Japanese Air Quality Standard was formulated to assess and compare air pollution levels among different areas. Ambient particulate matter with an aerodynamic diameter of 2.5 µm or less (PM_2.5_) and suspended particulate matter (SPM) are generally used as the indexes of air pollution. According to the Japanese Air Quality Standard, SPM is defined as particles with an aerodynamic with a diameter of less than 10 µm by the 100% cutoff point, which approximately corresponds to particulate matter <7 mm in aerodynamic diameter by the 50% cutoff point [4,5]. Studies have indicated that exposure to PM_2.5_ is associated with cardiopulmonary mortality [6-10], as well as with the development of asthma [11] and respiratory disease [12-14], while other studies have produced evidence that SPM is associated with respiratory health [5,15-17].

There have been limited epidemiological studies on the association between air pollutants and ocular diseases. Photochemical air pollution has been reported to have an effect on the conjunctival goblet cells [18] and to cause ocular irritation and discomfort [19]. In severe cases, air pollution has been reported to affect visual function [20]. Examination of a year-long dataset regarding air pollutants in Paris revealed a correlation between the degree of air pollution and a temporary surge in visits to an ophthalmology emergency department [21]. Exposure to high levels of air pollutants has various adverse effects on ocular surface health, such as photophobia, eye strain, irritation, dry eye, and watering [22]. Furthermore, elevated atmospheric concentrations of nitrogen dioxide (NO_2_) and nitric oxide (NO) impact the pH of tears, which diminishes as atmospheric sulfur dioxide (SO_2_) levels rise [23]. Air pollutants such as nitrogen oxides (NO_X_) and ozone (O_3_) have been reported to worsen symptoms related to allergic reactions in the nose and eyes [24]. Furthermore, an increase in integrated air pollutants such as PM_10_, O_3_, SO_2_, and NO_2_ has been reported to increase medical appointments for unspecified conjunctivitis at outpatient clinics [25].

However, very little has been published about the impact of air pollutants on the pathogenesis of allergic conjunctivitis [26]. We previously showed that the PM_2.5_ level is an independent predictor of outpatient visits for allergic conjunctivitis during the non-pollen season [27]. SPM may frequently induce a worsening of allergic conjunctivitis due to a stronger irritant effect compared with PM_2.5_ because SPM has a larger diameter. However, no epidemiological investigation of the influence of SPM on allergic conjunctivitis has been conducted. Accordingly, the present study aimed to investigate the influence of exposure to SPM on morbidity due to allergic conjunctivitis. For this purpose, we examined the relationship between the level of SPM and outpatient attendance rates for allergic conjunctivitis. The present report covers part of the results of our investigation into the association of PM_2.5_ with rates of outpatient visits related to allergic conjunctivitis [27].

## Materials and methods

Study region and survey of outpatient visits

This research received approval from the Institutional Review Board of Tokyo Women’s Medical University (#4161) and was conducted following the principles of the Helsinki Declaration. The investigation was conducted prospectively at Nerima West Eye Clinic (Tokyo, Japan) between January 1 and December 31, 2012. We documented the daily count of outpatient visits attributed to allergic conjunctivitis. Subsequently, we computed the mean number of outpatient visits per week for each week throughout the year and utilized these weekly averages for analysis. Allergic conjunctivitis was identified through clinical diagnosis in accordance with the published guidelines [28].

Observation of atmospheric circumstances

Information regarding atmospheric conditions was acquired from the Japan Meteorological Agency (http://www.jma.go.jp/jma/index.html) and the Japan Ministry of the Environment (http://www.env.go.jp/en/). The data on humidity, wind speed, and temperature, as well as the daily concentrations of total hydrocarbons (THC), non-methane hydrocarbons (NMH), methane (CH_4_), carbon monoxide (CO), NO_X_, NO_2_, NO, oxidants (O_X_), and SPM were obtained. According to reports from the Japan Meteorological Agency, concentrations of air quality factors were measured using either a high-performance liquid chromatograph-UV detector or a gas chromatograph-mass spectrometer.

Statistical analysis

Mean values were compared using the one-way analysis of variance and Scheffe’s multiple comparison test. To explore connections between continuous variables, Pearson’s two-tailed correlation coefficients were computed, and discrepancies between the correlation coefficients were assessed through Fisher’s Z transformation. Factors displaying a significant correlation with outpatient visits for allergic conjunctivitis were identified via forward stepwise multiple logistic regression analysis. Statistical significance was established at p-values <0.05. SAS System 9.1 software (SAS Institute Inc., Cary, North Carolina, USA) was utilized for statistical analysis.

## Results

During the study period, out of 30,749 patients visiting the clinic, 6,145 were diagnosed with allergic conjunctivitis. Table 1 presents the mean daily count of clinic visitors and the environmental factors categorized by each season. There were no substantial variations in the total daily clinic attendance across the four seasons. However, the environmental factors exhibited notable distinctions across seasons, as determined by one-way analysis of variance (p < 0.05).

**Table 1:** Comparison of outpatient counts and environmental variables across seasons. Data are shown as the mean ± standard deviation. *: One-way analysis of variance and Scheffe’s multiple comparison test. O_x_ = oxidants; NO = Nitric oxide; NO_2_ = nitrogen dioxide; NO_X_ = nitrogen oxides; CO = carbon monoxide; CH_4_ = methane; NMH = non-methane hydrocarbons; THC = total hydrocarbons; SPM = suspended particulate matter

Variable	Winter January–March	Spring April–June	Summer July–September	Autumn October–December	*P-value
Outpatients with allergic conjunctivitis	17.9 ± 7.7	16.4 ± 3.5	17.1 ± 3.5	14.9 ± 2.6	0.4781
O_x_ (ppb)	23.4 ± 8.4	38.8 ±8.0	25.4 ± 5.5	17.3 ± 5.3	<0.0001
NO (ppb)	8.1 ± 5.2	1.0 ± 0.5	2.1 ± 0.6	12.5 ± 10.7	<0.0001
NO_2_ (ppb)	20.4 ± 3.7	13.7 ± 2.0	12.2 ± 2.4	20.5 ± 4.3	<0.0001
NO_X_ (ppb)	28.5 ± 7.9	14.8 ± 2.4	14.2 ± 2.3	32.9 ± 14.2	<0.0001
CO (0.01ppm)	4.0 ± 0.5	3.0 ± 0.3	2.5 ± 0.6	4.1 ± 0.8	<0.0001
CH_4_ (0.01ppm)	197.9 ± 2.3	192.9 ± 1.4	188.1 ± 3.4	198.0 ± 2.9	<0.0001
NMH (0.01ppm)	19.1 ± 2.4	17.0 ± 1.6	17.2 ± 1.8	20.3 ± 3.4	0.0025
THC (0.01ppm)	217.0 ± 4.5	209.8 ± 2.6	205.3 ± 4.6	218.3 ± 6.1	<0.0001
Wind velocity (0.1 m/s)	13.6 ± 2.6	9.4 ± 3.9	8.5 ± 2.5	8.0 ± 2.9	<0.0001
Temperature (°C)	5.2 ± 2.6	17.6 ± 3.5	26.1 ± 2.3	10.3 ± 4.9	<0.0001
Humidity (%)	54.6 ± 9.4	69.1 ± 9.5	75.9 ± 4.6	65.3 ± 6.5	<0.0001
SPM (mg/m^3^)	18.3 ± 4.6	23.0 ± 4.3	24.3 ± 7.4	17.6 ± 3.2	0.0033

**Figure 1:**
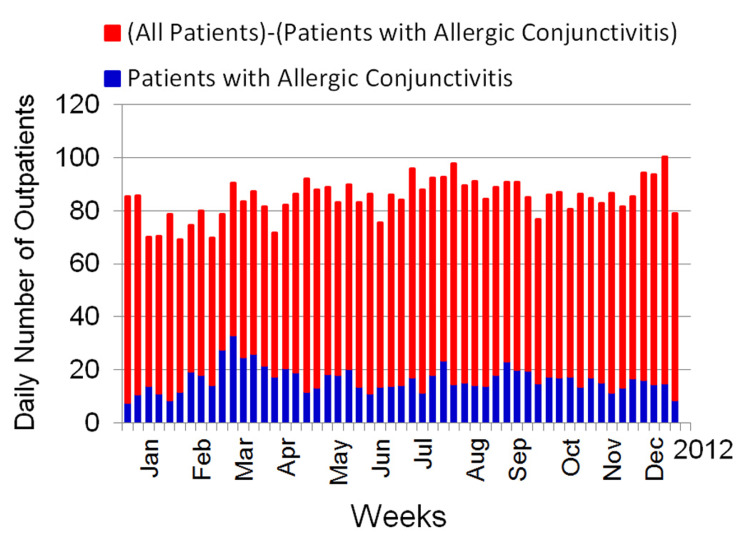
Seven-day average values for the total daily number of outpatients and the number of outpatients with allergic conjunctivitis.

The number of daily outpatient visits for allergic conjunctivitis varied greatly, spanning from 0 to 62. Figure 1 illustrates the mean weekly counts of total daily outpatients and those specifically for allergic conjunctivitis between January and December. Throughout this duration, three peaks in allergic conjunctivitis outpatient visits were evident, occurring during the final week of February, the closing week of July, and the initial week of September.

**Figure 2:**
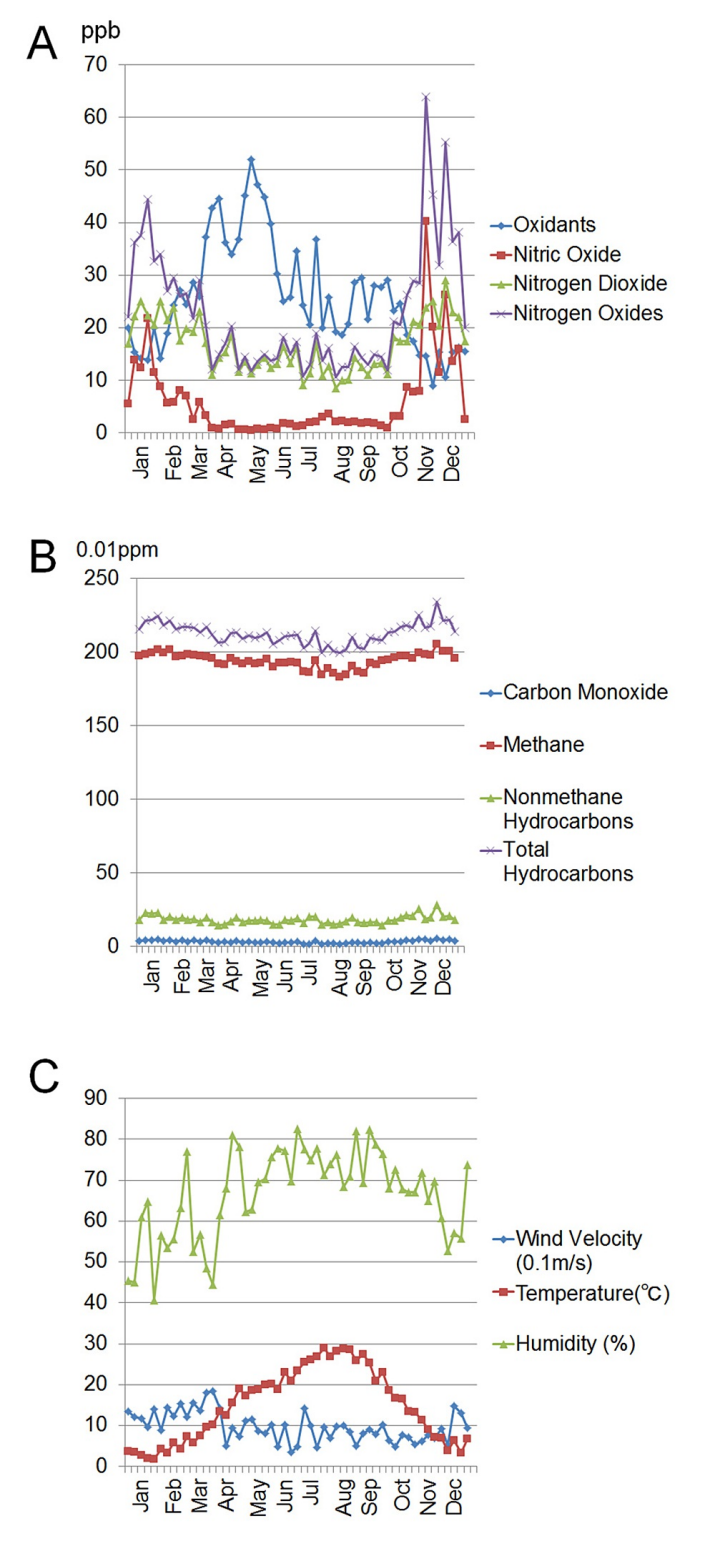
Seven-day average levels of air pollutants (A and B) and environmental parameters (C). (A) Atmospheric levels of oxidants, nitric oxide, nitrogen dioxide, and nitrogen oxide (ppb). (B) Atmospheric levels of carbon monoxide, methane, non-methane hydrocarbons, and total hydrocarbons (0.01 ppm). (C) Environmental parameters, including wind velocity (0.1 m/s), temperature (°C), and humidity (%).

**Figure 3:**
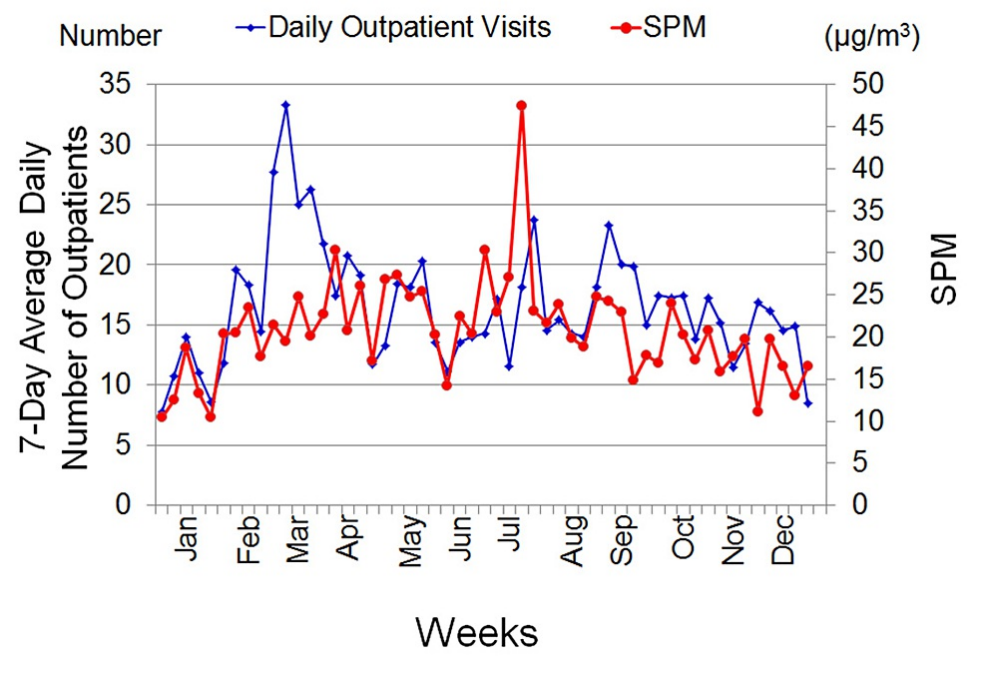
Seasonal changes in the number of outpatients with allergic conjunctivitis and the atmospheric level of SPM from January to December. SPM = suspended particulate matter

Figure 2 shows the seven-day average levels of each air pollutant and other parameters. Figure 3 demonstrates the relationship between the seven-day average level of SPM and the seven-day average values for the daily number of outpatients with allergic conjunctivitis. The level of SPM and the number of outpatients varied proportionally from January to February and April to May, and the number of outpatients also tended to vary with SPM after a delay of about one week from August to December (Figure 3). However, the number of outpatients changed independently of the SPM level during March, which is the beginning of the pollen season in Japan (Figure 3).

We divided the study period into four seasons (January to March, April to June, July to September, and October to December). As shown in Table 2, there was a positive correlation with the number of outpatients for the level of Ox (r = 0.70, p = 0.0077), temperature (r = 0.77, p = 0.0019), and SPM (r = 0.70, p = 0.0073) during winter from January to March, as well as a correlation with CO (r = 0.56, p = 0.0460) during spring from April to June (two-tailed Pearson’s correlation coefficient). Conversely, no associations were found between the outpatient count and any of the variables from July to December, covering the summer and autumn seasons (Table 2).

**Table 2:** Correlation between the number of patients with allergic conjunctivitis and environmental factors in each season. Correlations between weekly averages for the daily number of outpatients with allergic conjunctivitis in each season and the seven-day average levels of each parameter were calculated by the two-tailed Pearson’s product moment formula. R = two-tailed Pearson’s correlation coefficient; CI = confidence interval. O_x_: oxidants; NO = nitric oxide; NO_2_ = nitrogen dioxide; NO_x_ = nitrogen oxides; CO = carbon monoxide; CH_4_ = methane; NMH = non-methane hydrocarbons; THC = total hydrocarbons; SPM = suspended particulate matter

Correlation coefficient												
	January–March			April–June			July–September			October–December		
Variable	R	95% CI	P-value	R	95% CI	P-value	R	95% CI	P-value	R	95% CI	P-value
O_X_	0.70	0.24–0.90	0.0077	0.46	-0.12–0.81	0.1103	0.15	-0.42–0.63	0.6144	0.46	-0.12–0.81	0.1108
NO	-0.62	-0.87–0.11	0.0230	0.07	-0.50–0.60	0.8089	0.13	-0.43–0.62	0.6472	-0.31	-0.74–0.29	0.2980
NO_2_	-0.20	-0.67–0.40	0.5187	0.14	-0.44–0.64	0.6460	0.07	-0.48–0.58	0.8155	-0.15	-0.65–0.44	0.6213
NO_X_	-0.56	-0.85–0.01	0.0490	0.13	-0.45–0.63	0.6741	0.10	-0.46–0.60	0.7392	-0.28	-0.72–0.32	0.3484
CO	-0.43	-0.79–0.16	0.1400	0.56	0.01–0.85	0.0460	0.09	-0.46–0.60	0.7474	-0.35	-0.75–0.25	0.2457
CH_4_	-0.62	-0.87–0.11	0.0226	0.37	-0.22–0.77	0.2074	0.02	-0.52–0.54	0.9589	-0.10	-0.62–0.47	0.7351
NMH	-0.54	-0.84–0.01	0.0562	0.13	-0.45–0.64	0.6733	-0.33	-0.73–0.24	0.2441	-0.15	-0.65–0.43	0.6182
THC	-0.65	-0.88–0.15	0.0166	0.27	-0.33–0.71	0.3812	-0.12	-0.61–0.44	0.6930	-0.13	-0.63–0.46	0.6791
Wind velocity	0.48	-0.09–0.82	0.0944	0.40	-0.19–0.78	0.1731	-0.06	-0.57–0.48	0.8306	-0.29	-0.73–0.31	0.3280
Temperature	0.77	0.39–0.93	0.0019	-0.62	-0.87–0.11	0.0231	0.04	-0.50–0.56	0.8787	0.46	-0.12–0.81	0.1106
Humidity	0.32	-0.28–0.74	0.2804	-0.53	-0.84–0.03	0.0627	-0.04	-0.56–0.50	0.8835	-0.21	-0.68–0.38	0.4894
SPM	0.70	0.25–0.90	0.0073	0.53	-0.03–0.84	0.0618	0.01	-0.52–0.54	0.9658	0.20	-0.39–0.68	0.5135

**Figure 4:**
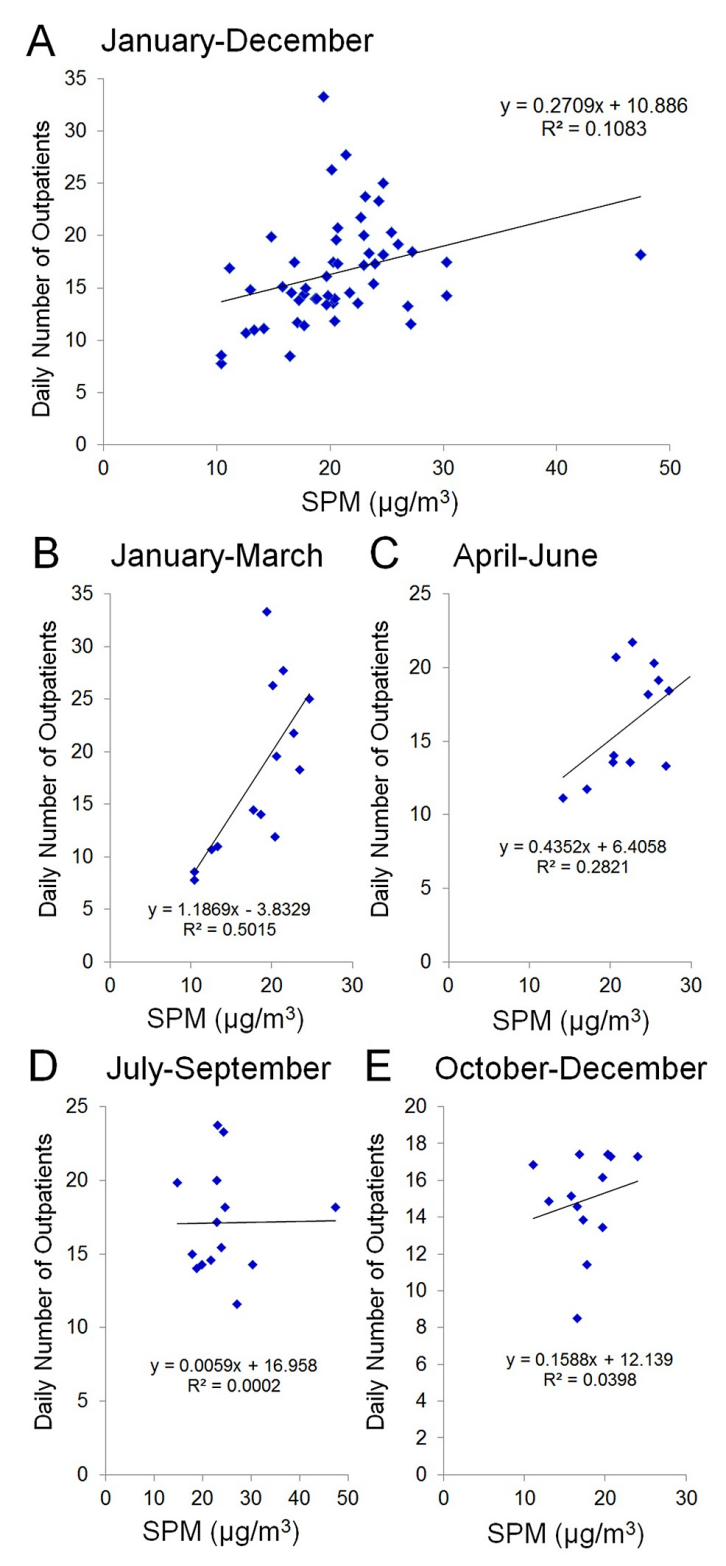
Relationship between the seven-day average daily number of outpatients with allergic conjunctivitis and the seven-day average PM2.5 level (mg/m3). (A) Relationship from January to December. (B) Relationship from January to March. (C) Relationship from April to June. (D) Relationship from July to September. (E) Relationship from October to December. The horizontal axis is the concentration of SPM, and the vertical axis indicates the number of patients. SPM = suspended particulate matter

The outpatient count increased concurrently with the escalation of SPM from January through June. (Figures 4A-4C), but there was no relationship between July and December (Figures 4D, 4E).

**Figure 5:**
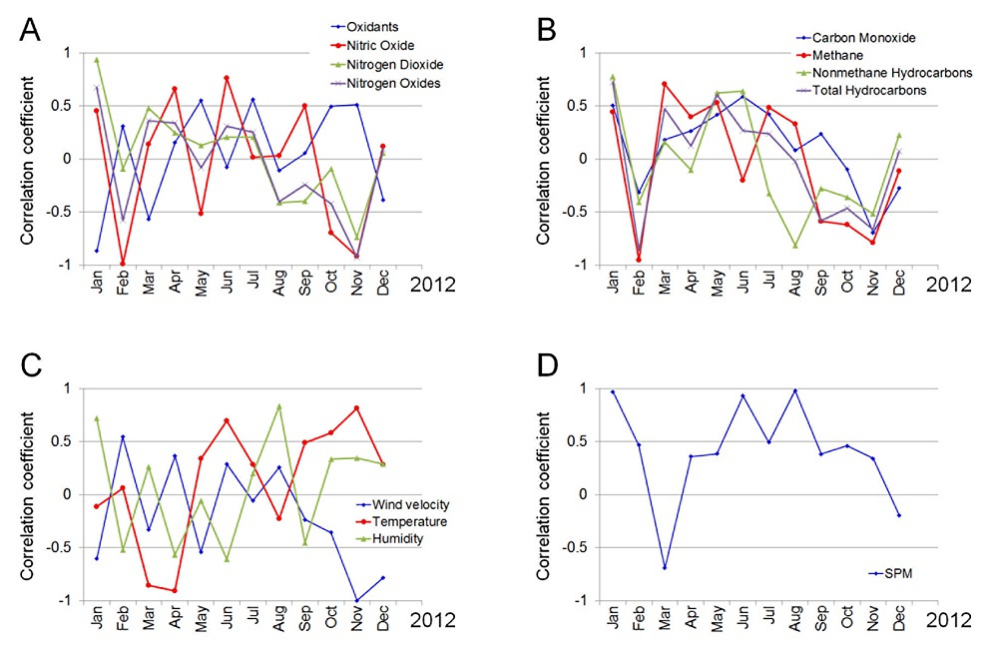
Seasonal changes of the correlations between the seven-day average daily number of outpatients with allergic conjunctivitis and the seven-day average levels of air pollutants and environmental parameters. (A) Variation in the correlation coefficient between the concentration of oxidants, nitric oxide, nitrogen dioxide, and nitrogen oxides and the number of patients. (B) Variation in the correlation coefficient between the concentration of carbon monoxide, methane, non-methane hydrocarbons, and total hydrocarbons and the number of patients. (C) Variation in the correlation coefficient between wind velocity, temperature, and humidity and the number of patients. (D) Variation in the correlation coefficient between SPM and the number of patients.

Figure 5 shows seasonal changes in the correlation between the seven-day average daily number of outpatients with allergic conjunctivitis and the seven-day average levels of air pollutants and environmental parameters. Correlation coefficients for the relationship between daily outpatients with allergic conjunctivitis and the level of SPM were above 0.3 in every month except for March and December. On the other hand, correlations of the other parameters with outpatient visits varied greatly during the study period.

Table 3 displays correlations between selected variables and the number of outpatients with allergic conjunctivitis (two-tailed Pearson’s product moment formula). The number of outpatients with allergic conjunctivitis was positively correlated with O_X_ (r = 0.33, p = 0.0172) and SPM (r = 0.33, p = 0.0161), while it was negatively correlated with NO (r = -0.28, p = 0.0430). The multivariate analysis indicated a connection between the count of allergic conjunctivitis-related outpatients and the wind speed (odds ratio (OR) = 1.52, p = 0.0038) and SPM level (OR = 1.37, p = 0.0038).

**Table 3:** Correlation coefficients and multivariate odds ratios for predictors of the number of outpatients from January to December 2012. Correlations between weekly averages for the daily number of outpatients with allergic conjunctivitis and the seven-day average levels of each parameter were calculated by the two-tailed Pearson’s product moment formula. R = two-tailed Pearson’s correlation coefficient. Independent determinants of the number of outpatients with allergic conjunctivitis were investigated by multiple logistic regression analysis. OR = odds ratio. O_x_: oxidants; NO = nitric oxide; NO_2_ = nitrogen dioxide; NO_x_ = nitrogen oxides; CO = carbon monoxide; CH_4_ = methane; NMH = non-methane hydrocarbons; THC = total hydrocarbons; SPM = suspended particulate matter

		Correlation coefficient		Multivariate analysis	
Variable	R	95% CI	P-value	OR	P-value
O_X_	0.33	0.06–0.55	0.0172	–	–
NO	-0.28	-0.51–0.01	0.0430	–	–
NO_2_	-0.08	-0.34–0.19	0.5613	–	–
NO_X_	-0.22	-0.46–0.06	0.1164	–	–
CO	-0.13	-0.38–0.15	0.3625	–	–
CH_4_	-0.13	-0.39–0.15	0.3593	–	–
NMH	-0.26	-0.50–0.01	0.0596	–	–
THC	-0.19	-0.44–0.08	0.1664	–	–
Wind velocity	0.24	-0.03–0.48	0.0815	1.52	0.0038
Temperature	0.09	-0.19–0.35	0.5229	–	–
Humidity	-0.01	0.28–0.26	0.9430	–	–
SPM	0.33	0.06–0.55	0.0161	1.37	0.0161

Correlations were also investigated among pollutants and environmental parameters during the study period (Table 4). The level of SPM was positively correlated with that of O_X_ (r = 0.51, p < 0.0001), as well as with temperature (r = 0.49, p=0.0002) and humidity (r = 0.37, p = 0.0063), while it was negatively correlated with NO (r = -0.37, p = 0.0059), NOx (r = -0.35, p = 0.0099), CH_4_ (r = -0.36, p = 0.0089), and THC (r = -0.30, p = 0.0319) (Table 4).

**Table 4:** Pearson’s correlations for seven-day average levels of daily pollutant concentrations in Tokyo from January to December 2012. Correlation coefficients were calculated by the two-tailed Pearson’s product moment formula. *P < 0.05. O_x_: oxidants; NO = nitric oxide; NO_2_ = nitrogen dioxide; NO_x_ = nitrogen oxides; CO = carbon monoxide; CH_4_ = methane; NMH = non-methane hydrocarbons; THC = total hydrocarbons; SPM = suspended particulate matter

	O_X_	NO	NO_2_	NO_X_	CO	CH_4_	NMH	THC	Wind Velocity	TP	Humidity
NO	-0.61*										
NO_2_	-0.54*	0.73*									
NO_x_	-0.63*	0.96*	0.90*								
CO	-0.40*	0.74*	0.93*	0.88*							
CH_4_	-0.32*	0.64*	0.89*	0.79*	0.90*						
NMH	-0.50*	0.78*	0.79*	0.84*	0.78*	0.68*					
THC	-0.42*	0.75*	0.92*	0.88*	0.93*	0.96*	0.86*				
Wind velocity	0.10	-0.01	0.07	0.03	0.11	0.11	-0.18	0.01			
Temperature	0.35*	-0.57*	-0.82*	-0.72*	-0.83*	-0.89*	-0.51*	-0.82*	-0.42*		
Humidity	0.15	-0.35*	-0.48*	-0.44*	-0.48*	-0.54*	-0.25*	-0.48*	-0.61*	0.72*	
SPM	0.51*	-0.37*	-0.25	-0.35*	-0.20	-0.36*	-0.11	-0.30*	-0.17	0.49*	0.37*

## Discussion

The relationship between allergic conjunctivitis and SPM has not been investigated previously. This study demonstrated that the number of patients with allergic conjunctivitis was correlated with the level of SPM in a hospital-based population in January-March and April-June. The count of allergic conjunctivitis outpatients exhibited a noteworthy correlation with heightened levels of SPM in the atmosphere. This relationship appeared more pronounced during winter and spring compared to summer and fall. Notably, the multivariate analysis indicated that among multiple factors considered, only SPM emerged as a significant determinant influencing the number of allergic conjunctivitis outpatient cases throughout the study period. These analyses strongly imply an association between SPM and the exacerbation of allergic conjunctivitis symptoms.

In this study, secondary data were collected for atmospheric parameters. The advantage of using secondary data is that it is less expensive than primary information and allows the necessary information to be collected in a shorter time. On the other hand, the reliability of secondary information may affect the results of statistical processing. In this study, data strictly measured by the Japan Meteorological Agency and the Japan Ministry of the Environment were used. Therefore, the secondary data used in this study did not affect the statistical processing and results.

Recently, several studies have addressed the association between SPM and cardiovascular or respiratory diseases [17]. PM_2.5_ and PM_10_ have both been demonstrated to be associated with respiratory symptoms in asthma patients [29-35]. Additionally, studies have demonstrated the impact of SO_2_, NO_2_, and O_3_ on individuals with asthma [36,37]. However, to our understanding, there has been minimal exploration into the prevalence of SPM concerning allergic conjunctivitis. Conversely, assessments have been made regarding the correlation between air pollutants and ocular surface diseases. For instance, studies have highlighted air pollutants such as PM_10_, O_3_, SO_2_, and NO_2_ as contributors to escalated outpatient visits due to non-specific conjunctival disorders [25]. Our study showed a positive association between SPM and outpatient visits for allergic conjunctivitis (r = 0.70 in winter, and r = 0.53 in spring). Previously, we also demonstrated that PM_2.5_ is a predictor of outpatient visits for allergic conjunctivitis during the non-pollen season [27]. Conversely, studies have indicated that PM_2.5_ has a relatively minor impact on the occurrence of ocular surface diseases when compared to PM_10_, O_3_, SO_2_, and NO_2_ [25]. PM_2.5_ has a smaller diameter than SPM or PM_10_, and larger particles may cause a stronger inflammatory response of the conjunctiva than smaller particles.

Our study demonstrated a significant positive correlation between the level of O_x_ and outpatient visits for allergic conjunctivitis during winter and spring (r = 0.70 in winter, r = 0.46 in spring). O_x_ in photochemical smog includes O_3_, hydrogen peroxide, NO_2_, and nitric acid. Research conducted by Riediker et al. found that O_3_ and NO_x _can worsen allergic rhinoconjunctivitis symptoms [24]. O_3_ serves as a potent oxidizing agent and is frequently utilized in industrial settings for oxidation procedures, as well as in urban areas for purifying tap water. Furthermore, atmospheric O_3_ is a significant constituent of photochemical smog. Because of its strong oxidative potential, O_3_ can damage the human nasal mucosa [38] and conjunctival mucosa [24,25]. Furthermore, the presence of O_x_ (including SO, NO_2_, and NO) in the air alters the composition of the tear fluid, rendering it acidic [23]. Consequently, this acidified tear fluid affected by O_x_ can lead to the inflammation of cells present on the surface of the conjunctiva.

During the spring season, a notable positive correlation was observed between outpatient visits for allergic conjunctivitis and CO levels (r = 0.56 in spring). Norris et al. reported that PM and CO were related to daily emergency department visits for asthma [39]. Our study also showed a positive correlation between CO and other pollutants such as NO (r = 0.74), NO_2_ (r = 0.93), NO_x_ (r = 0.88), CH_4_ (r = 0.90), NMH (r = 0.78), and THC (r = 0.93) (Table 4). However, we found that CO was negatively correlated with Ox (r = -0.4) and SPM (r = -0.20). In this study, O_x_ and SPM were the two major predictors of outpatient visits for allergic conjunctivitis. Because there is no evidence that CO induces conjunctival damage, CO would be expected to have little influence on allergic conjunctivitis.

The curve of variation in the number of patients with allergic conjunctivitis was very similar to the curve of variation in the level of SPM in the air, with statistically significant differences (Table 3) by multivariate analysis, and a significant correlation was observed between outpatient visits and SPM (r = 0.33, p = 0.0161). There are multiple reasons why SPM induces a more pronounced conjunctival inflammation compared to other air pollutants. First, SPM can act as an adjuvant for allergic sensitization in patients with allergic conjunctivitis. It has been reported that SPM has an adjuvant effect on the production of IgE targeting common environmental allergens [40]. Four molds (Fusarium, Aspergillus, Penicillium, and Basipetospora) were identified in samples of SPM in western Korea [41]. Furthermore, the spores of *Aquabacterium*, *Flavobacteriales*, and *Prevotellaceae* were detected in airborne dust in Korea [42]. Moreover, airborne dust in southern Taiwan has been found to contain various molds [43]. These data suggest that SPM contains various sensitizing allergens and pollen and fungi attached to SPM may cause allergic conjunctivitis. The second reason why SPM may provoke conjunctivitis is the physical form in the atmosphere at standard temperature and pressure. SPM are solid dust particles, while other air pollutants (including Ox, NO, NO_2_, NOx, CO, CH_4_, NMH, and THC) are gases. Thus, SPM can induce inflammation related to friction with the conjunctiva, while gaseous air pollutants are unlikely to have such an effect. SPM may also alter tear film stability, causing break-up and thinning of the tear film by disrupting the tear lipid layer.

Next, we examined the relationship between the number of outpatients and the level of air pollution in each season. The number of patients was significantly correlated with the level of SPM in winter (r = 0.70), but there was no relationship in spring (r = 0.53), summer (r = 0.01), or autumn (r = 0.20) (Table 2). One possible reason is that atmospheric pollen levels increase during spring and autumn in Japan as pollen is released from the forests twice per year [44-48], while house dust mites and mold are the main causes of seasonal allergy in the humid summer and autumn months. Because our study did not assess outdoor allergens such as pollen and mites, our results might have been influenced by airborne allergens (pollen and mites) as well as air pollutants during spring, summer, and autumn.

There were several limitations of this study, as we reported in our previous study [27]. The onset of allergic conjunctivitis and outpatient attendance may be delayed after the increase in SPM levels. We also did not examine the age distribution of patients with allergic conjunctivitis. Furthermore, this study did not examine the effects of exposure to house dust or mold in the home. We also did not use data on past allergic history or occupational history factors in the statistical processing and calculations. The background of these patients should also be examined in the future.

## Conclusions

We observed a significant association between the daily number of outpatients with allergic conjunctivitis attending a hospital in Tokyo and the airborne level of SPM. This suggests a possible role of SPM in the development of allergic conjunctivitis. An association between SPM and allergic conjunctivitis could have broad public health implications in relation to the management of allergic diseases.

## References

[1] Tomin no kenkou to anzen wo kakuho suru kankyo ni kansuru jyourei (Tokyo-to Jyourei Dai 215 Gou) (in Japanese); Tokyo Metropolitan Government (2014). Tomin no kenkou to anzen wo kakuho suru kankyo ni kansuru jyourei (Tokyo-to Jyourei Dai 215 Gou) (in Japanese). December.

[2] Yamamoto N, Muramoto A, Yoshinaga J (2007). Comparison of carbonaceous aerosols in Tokyo before and after implementation of diesel exhaust restrictions. Environ Sci Technol.

[3] Hara K, Homma J, Tamura K, Inoue M, Karita K, Yano E (2013). Decreasing trends of suspended particulate matter and PM2.5 concentrations in Tokyo, 1990-2010. J Air Waste Manag Assoc.

[4] Katanoda K, Sobue T, Satoh H (2011). An association between long-term exposure to ambient air pollution and mortality from lung cancer and respiratory diseases in Japan. J Epidemiol.

[5] Yoda Y, Otani N, Sakurai S, Shima M (2014). Acute effects of summer air pollution on pulmonary function and airway inflammation in healthy young women. J Epidemiol.

[6] Laden F, Schwartz J, Speizer FE, Dockery DW (2006). Reduction in fine particulate air pollution and mortality: extended follow-up of the Harvard Six Cities study. Am J Respir Crit Care Med.

[7] Ostro B, Broadwin R, Green S, Feng WY, Lipsett M (2006). Fine particulate air pollution and mortality in nine California counties: results from CALFINE. Environ Health Perspect.

[8] Pope CA 3rd, Dockery DW (2006). Health effects of fine particulate air pollution: lines that connect. J Air Waste Manag Assoc.

[9] Samoli E, Peng R, Ramsay T (2008). Acute effects of ambient particulate matter on mortality in Europe and North America: results from the APHENA study. Environ Health Perspect.

[10] Huang W, Cao J, Tao Y (2012). Seasonal variation of chemical species associated with short-term mortality effects of PM(2.5) in Xi'an, a Central City in China. Am J Epidemiol.

[11] Ward DJ, Roberts KT, Jones N, Harrison RM, Ayres JG, Hussain S, Walters S (2002). Effects of daily variation in outdoor particulates and ambient acid species in normal and asthmatic children. Thorax.

[12] Schwartz J, Neas LM (2000). Fine particles are more strongly associated than coarse particles with acute respiratory health effects in schoolchildren. Epidemiology.

[13] Hrubá F, Fabiánová E, Koppová K, Vandenberg JJ (2001). Childhood respiratory symptoms, hospital admissions, and long-term exposure to airborne particulate matter. J Expo Anal Environ Epidemiol.

[14] Yap PS, Gilbreath S, Garcia C, Jareen N, Goodrich B (2013). The influence of socioeconomic markers on the association between fine particulate matter and hospital admissions for respiratory conditions among children. Am J Public Health.

[15] Odajima H, Yamazaki S, Nitta H (2008). Decline in peak expiratory flow according to hourly short-term concentration of particulate matter in asthmatic children. Inhal Toxicol.

[16] Ueda K, Nitta H, Odajima H (2010). The effects of weather, air pollutants, and Asian dust on hospitalization for asthma in Fukuoka. Environ Health Prev Med.

[17] Hasunuma H, Ishimaru Y, Yoda Y, Shima M (2014). Decline of ambient air pollution levels due to measures to control automobile emissions and effects on the prevalence of respiratory and allergic disorders among children in Japan. Environ Res.

[18] Waheed MA, Basu PK (1970). The effect of air pollutants on the eye. I. The effect of an organic extract on the conjunctival goblet cells. Can J Ophthalmol.

[19] Altshuller AP (1977). Eye irritation as an effect of photochemical air pollution. J Air Pollut Control Assoc.

[20] Basu PK (1972). Air pollution and the eye. Surv Ophthalmol.

[21] Bourcier T, Viboud C, Cohen JC (2003). Effects of air pollution and climatic conditions on the frequency of ophthalmological emergency examinations. Br J Ophthalmol.

[22] Gupta SK, Gupta SC, Agarwal R, Sushma S, Agrawal SS, Saxena R (2007). A multicentric case-control study on the impact of air pollution on eyes in a metropolitan city of India. Indian J Occup Environ Med.

[23] Andrés S, García ML, Espina M, Valero J, Valls O (1988). Tear pH, air pollution, and contact lenses. Am J Optom Physiol Opt.

[24] Riediker M, Monn C, Koller T, Stahel WA, Wüthrich B (2001). Air pollutants enhance rhinoconjunctivitis symptoms in pollen-allergic individuals. Ann Allergy Asthma Immunol.

[25] Chang CJ, Yang HH, Chang CA, Tsai HY (2012). Relationship between air pollution and outpatient visits for nonspecific conjunctivitis. Invest Ophthalmol Vis Sci.

[26] Fujishima H, Satake Y, Okada N, Kawashima S, Matsumoto K, Saito H (2013). Effects of diesel exhaust particles on primary cultured healthy human conjunctival epithelium. Ann Allergy Asthma Immunol.

[27] Mimura T, Ichinose T, Yamagami S (2014). Airborne particulate matter (PM2.5) and the prevalence of allergic conjunctivitis in Japan. Sci Total Environ.

[28] Ben Ezra D (1994). Guidelines on the diagnosis and treatment of conjunctivitis. Ocul Immunol Inflam.

[29] Delfino RJ, Zeiger RS, Seltzer JM, Street DH, McLaren CE (2002). Association of asthma symptoms with peak particulate air pollution and effect modification by anti-inflammatory medication use. Environ Health Perspect.

[30] Delfino RJ, Quintana PJ, Floro J (2004). Association of FEV1 in asthmatic children with personal and microenvironmental exposure to airborne particulate matter. Environ Health Perspect.

[31] Rabinovitch N, Strand M, Gelfand EW (2006). Particulate levels are associated with early asthma worsening in children with persistent disease. Am J Respir Crit Care Med.

[32] Hansel NN, Breysse PN, McCormack MC (2008). A longitudinal study of indoor nitrogen dioxide levels and respiratory symptoms in inner-city children with asthma. Environ Health Perspect.

[33] McCormack MC, Breysse PN, Matsui EC (2009). In-home particle concentrations and childhood asthma morbidity. Environ Health Perspect.

[34] Breysse PN, Diette GB, Matsui EC, Butz AM, Hansel NN, McCormack MC (2010). Indoor air pollution and asthma in children. Proc Am Thorac Soc.

[35] Weinmayr G, Romeo E, De Sario M, Weiland SK, Forastiere F (2010). Short-term effects of PM10 and NO2 on respiratory health among children with asthma or asthma-like symptoms: a systematic review and meta-analysis. Environ Health Perspect.

[36] Koren HS (1995). Associations between criteria air pollutants and asthma. Environ Health Perspect.

[37] Jerrett M, Shankardass K, Berhane K (2008). Traffic-related air pollution and asthma onset in children: a prospective cohort study with individual exposure measurement. Environ Health Perspect.

[38] Pacini S, Giovannelli L, Gulisano M (2003). Association between atmospheric ozone levels and damage to human nasal mucosa in Florence, Italy. Environ Mol Mutagen.

[39] Norris G, YoungPong SN, Koenig JQ, Larson TV, Sheppard L, Stout JW (1999). An association between fine particles and asthma emergency department visits for children in Seattle. Environ Health Perspect.

[40] Ormstad H (2001). [Airborne dust particles in indoor environment and allergy]. Tidsskr Nor Laegeforen.

[41] Yeo HG, Kim JH (2001). SPM and fungal spores in the ambient air of west Korea during the Asian dust (yellow sand) period. Atmos Environ.

[42] Lee S, Choi B, Yi SM, Ko G (2009). Characterization of microbial community during Asian dust events in Korea. Sci Total Environ.

[43] Wu PC, Tsai JC, Li FC, Lung SC, Su HJ (2004). Increased levels of ambient fungal spores in Taiwan are associated with dust events from China. Atmos Environ.

[44] Fujishima H, Sahashi N, Shimazaki J, Tsubota K (1995). Allergic conjunctivitis caused by sugi (Cryptomeria japonica D. Don) pollen out of season. Asian Pac J Allergy Immunol.

[45] Mimura T, Amano S, Funatsu H (2004). Correlations between allergen-specific IgE serum levels in patients with allergic conjunctivitis in spring. Ocul Immunol Inflamm.

[46] Mimura T, Yamagami S, Amano S (2005). Allergens in Japanese patients with allergic conjunctivitis in autumn. Eye (Lond).

[47] Mimura T, Usui T, Mori M, Funatsu H, Noma H, Amano S (2011). Specific tear IgE in patients with moderate-to-severe autumnal allergic conjunctivitis. Int Arch Allergy Immunol.

[48] Mimura T, Usui T, Yamagami S, Miyai T, Amano S (2013). Relationship between total tear IgE and specific serum IgE in autumnal allergic conjunctivitis. Cornea.

